# Sex matters in CSU: Women face greater burden and poorer urticaria control, especially in midlife—CURE insights

**DOI:** 10.1111/jdv.70027

**Published:** 2025-09-18

**Authors:** Emek Kocatürk, Pascale Salameh, Riccardo Asero, Mojca Bizjak, Ana Gimenez‐Arnau, Clive Grattan, David Pesqué, Nidia Planella‐Fontanillas, Leonie Shirin Herzog, Thomas Buttgereit, Hanna Bonnekoh, Daria Fomina, Elena Kovalkova, Marina Lebedkina, Alicja Kasperska‐Zajac, Magdalena Zając, Mateusz Zamłyński, Kanokvalai Kulthanan, Papapit Tuchinda, Maryam Khoshkhui, Zohreh Hassanpour, Jonny Peter, Aurelie Du‐Thanh, Raisa Meshkova, Mohamed Abuzakouk, Michael Makris, Laurence Bouillet, Alexis Bocquet, Stamatios Gregoriou, Simon Francis Thomsen, Joachim Dissemond, Petra Staubach, Andrea Bauer, Inna Danilycheva, Martijn van Doorn, Claudio Parisi, Martin Metz, Joachim W. Fluhr, Torsten Zuberbier, Karsten Weller, Pavel Kolkhir

**Affiliations:** ^1^ Institute of Allergology, Charité – Universitätsmedizin Berlin, Corporate Member of Freie Universität Berlin and Humboldt‐Universität zu Berlin Berlin Germany; ^2^ Fraunhofer Institute for Translational Medicine and Pharmacology ITMP, Immunology and Allergology Berlin Germany; ^3^ Department of Dermatology Bahçeşehir University School of Medicine Istanbul Turkey; ^4^ Gilbert and Rose‐Marie Chagoury School of Medicine Lebanese American University Beirut Lebanon; ^5^ Department of Primary Care and Population Health University of Nicosia Medical School Nicosia Cyprus; ^6^ Faculty of Pharmacy Lebanese University Hadat Lebanon; ^7^ Institut National de Santé Publique d’Épidémiologie Clinique et de Toxicologie‐Liban (INSPECT‐LB) Beirut Lebanon; ^8^ Ambulatorio di Allergologia, Clinica San Carlo Paderno Dugnano Italy; ^9^ Division of Allergy University Clinic of Respiratory and Allergic Diseases Golnik Golnik Slovenia; ^10^ Department of Dermatology Hospital del Mar Research Institute, Universitat Pompeu Fabra Barcelona Spain; ^11^ Guy’s Hospital, St John’s Institute of Dermatology London UK; ^12^ Moscow Research and Clinical Center of Allergy and Immunology, Moscow Healthcare Department City Clinical Hospital Moscow Russia; ^13^ Department of Clinical Immunology and Allergology I.M. Sechenov First Moscow State Medical University (Sechenov University) Moscow Russia; ^14^ Department of Pulmonology Astana Medical University Astana Kazakhstan; ^15^ European Center for Diagnosis and Treatment of Urticaria/Angioedema (GA2LEN UCARE/ACARE Network), Department of Clinical Allergology and Urticaria Medical University of Silesia Katowice Poland; ^16^ Department of Dermatology, Faculty of Medicine Siriraj Hospital Mahidol University Bangkok Thailand; ^17^ Allergy Research Center Mashhad University of Medical Sciences Mashhad Iran; ^18^ Department of Allergy and Immunology, School of Medicine Jundishapur University of Medical Sciences Ahvaz Iran; ^19^ Division of Allergy and Clinical Immunology, University of Cape Town, Allergy and Immunology Unit University of Cape Town Lung Institute Cape Town South Africa; ^20^ Dermatology Department University of Montpellier Montpellier France; ^21^ Department of Clinical Immunology and Allergology Smolensk State Medical University Smolensk Russian Federation; ^22^ Department of Allergy & Immunology, MSI Cleveland Clinic Abu Dhabi UAE; ^23^ Allergy Unit “D. Kalogeromitros”, 2nd Department Dermatology and Venereology National and Kapodistrian University of Athens, “Attikon” University Hospital Athens Greece; ^24^ National Reference Center for Angioedema Grenoble University Hospital Grenoble France; ^25^ 1st Department of Dermatology‐Venereology Andreas Sygros Hospital, National and Kapodistrian University of Athens Athina Greece; ^26^ Department of Biomedical Sciences Bispebjerg Hospital Copenhagen Denmark; ^27^ Department of Dermatology University of Copenhagen Copenhagen Denmark; ^28^ Department of Dermatology, Venerology and Allergology University of Essen Essen Germany; ^29^ Department of Dermatology University Medical Center Mainz Germany; ^30^ Department of Dermatology University Hospital, Carl Gustav Carus, Technical University Dresden Dresden Germany; ^31^ Department of Allergology NRC Institute of Immunology FMBA of Russia Moscow Russia; ^32^ Department of Dermatology Erasmus MC Rotterdam the Netherlands; ^33^ Centre for Human Drug Research Leiden the Netherlands; ^34^ Hospital Italiano de Buenos Aires, Secciones Allergia Buenos Aires Argentina

**Keywords:** age, angioedema, autoimmune disease, chronic disease, chronic spontaneous urticaria, comorbidity, depression, female, health status disparities, hormones, male, menopause, microchimerism, quality of life, sex differences, treatment outcome, urticaria

## Abstract

**Background:**

Chronic spontaneous urticaria (CSU), a disease predominantly affecting females, has limited information available on its differences between females and males of varying ages.

**Objectives:**

To investigate sex differences in age groups regarding disease activity, comorbidities, quality of life (QoL) and treatment patterns in CSU patients.

**Methods:**

We analysed Chronic Urticaria Registry (CURE) data, an international real‐world registry for patients with chronic urticaria. Patients were recruited via an online platform using a standardized questionnaire. The data were analysed for demographics, age of onset, duration of urticaria, (Urticaria Activity Score [UAS], Urticaria Control Test [UCT], Chronic Urticaria Quality of Life Questionnaire [CU‐Q2oL]), family history, systemic symptoms, aggravating factors, comorbidities, smoking and alcohol consumption, laboratory parameters, burden of disease, treatment distribution and response rates, compliance to treatment and adverse events. Comparisons were made among age groups <13, 13–17, 18–30, 31–50, 51–65 and >65 years.

**Results:**

Across 4136 CSU patients (from 58 sites across 29 countries), 2994 (72.4%) were female. Statistically significant female predominance started at age 31 (<0.001). Compared with males, females showed higher rates of angioedema (59.6 vs. 51.7%; *p* < 0.001), systemic symptoms (34.6 vs. 25.4%; *p* < 0.001), sleep disturbance (38.9 vs. 32.5%; *p* < 0.001), QoL impairment (CU‐Q2oL score 32 vs. 27.7; *p* < 0.001) and lower rates of urticaria control than males in all medication categories (*p* < 0.05 for all). Females had more concomitant diseases, including asthma, thyroid disease, obesity, autoimmune disease, gastrointestinal disease and depression (*p* < 0.05 for all). The disease was especially more burdensome and refractory in females aged 51–65 years than males, evidenced by more angioedema and systemic symptoms, worse QoL, lower UCT scores and more emergency visits (*p* < 0.05 for all). However, these differences were not prominent in the elderly females (>65 years).

**Conclusions:**

Compared with males, female CSU patients experience more burdensome disease, which gets worse in midlife.

Clinicaltrials.gov (or equivalent) listing (if applicable None).

## Graphical abstract

Chronic spontaneous urticaria affects more females than males, with predominance starting at age 31. The rates of several symptoms, including angioedema, sleep disturbance and systemic symptoms, are significantly higher in females than in males. Overall, females face greater burden and poorer urticaria control.

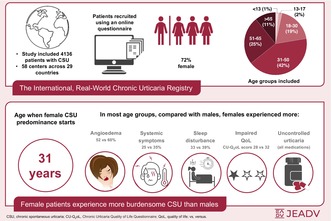

**Why was the study undertaken?**

- Differences in clinical presentation, comorbidities and treatment responses between male and female patients with chronic spontaneous urticaria (CSU) across age groups were investigated to gain clearer insights into how female CSU differs from male CSU and how hormonal transitions in females contribute to their distinct disease burden compared to males.

**What does this study add?**

- It is the largest real‐world analysis to date comparing female and male CSU patients.
- Female predominance in CSU emerges at age 31
- Women—especially in mid‐life—have more severe, treatment‐refractory CSU marked by higher angioedema rates, poorer UCT scores, greater QoL impairment and more emergency visits.
- Females have more comorbid conditions compared to males, and these tend to appear after age 30.
- The differences between female and male CSU patients mainly disappear after age 65.

**What are the implications of this study for disease understanding and/or clinical care?**

- Our findings suggest that the increased burden and refractoriness of CSU in females, particularly during midlife, may be driven by a complex interplay of hormonal transitions, systemic inflammation, immune dysregulation, and autoimmune comorbidities—supporting a distinct, possibly Type IIb autoimmune endotype more prevalent in women.
- The study underscores the clinical need for sex‐ and age‐tailored management: routine screening for thyroid/autoimmune, metabolic and mental‐health comorbidities in women aged 31–65 and early escalation of therapy when control is poor.

## INTRODUCTION

Chronic spontaneous urticaria (CSU) represents a long‐standing challenge in dermatological practice.^1^ It is characterized by wheals, angioedema, or both that persist for over 6 weeks.^2^ The condition notably deteriorates patients' quality of life (QoL), affecting social interactions, intimate relationships and work productivity.^3^

CSU is a mast cell‐mediated disease, and its underlying mechanism is based on autoimmunity, driven by two distinct pathways of mast cell activation: the Type IIb autoimmune endotype (caused by IgG‐type autoantibodies against the FcER1 and IgE) and the Type I autoallergic endotype (caused by IgE‐type autoantibodies against self‐antigens such as thyroid peroxidase).^4^

CSU demonstrates a pronounced sex disparity, with females being disproportionately affected—evidenced by at least twofold higher incidences in females compared with males. This disparity is consistent across adult populations with chronic urticaria (CU).^5^, ^6^, ^7^, ^8^ Intriguingly, the onset of female predominance from the age of 15 years suggests that developmental and hormonal factors may play a significant role in the manifestation and progression of CSU.^5^

The Chronic Urticaria Registry (CURE) is an ongoing, international, multicentre, observational, CU patient registry designed to improve scientific understanding and healthcare in patients with CU. CURE offers the perfect data source to study specific aspects of CU, such as sex disparities between females and males. Epidemiological data reveal that females not only have a higher incidence of CSU but also suffer from a more severe disease course. The association between being female and experiencing severe CSU of longer disease duration with a worse prognosis highlights the unique burden shouldered by this demographic.^9^ Additionally, female patients experience more angioedema, greater sleep disturbances, sexual dysfunction, fatigue and overall QoL impairment.^10^, ^11^, ^12^, ^13^, ^14^, ^15^, ^16^

Modern theories suggest that the higher incidence of autoimmune diseases in women is not solely driven by hormonal differences, as once thought, but also by sex‐specific genetic mechanisms—particularly the incomplete inactivation of immune‐related genes on the X chromosome.^17^ Since sex affects molecular and cellular processes, it can potentially affect clinical features and responses to treatments in chronic diseases. It is not known if particular triggers or aggravators are more common in female patients with CSU, if there are specific clinical features of CSU exclusive to one, or if females and males with CSU respond differently to particular treatments and if these differences change among different age groups.

While some differences between sexes in CSU have been acknowledged, previous studies are small and limited to specific countries or populations and they were not intended to directly compare features between females and males with CSU, leaving gaps in our understanding of the full scope of these differences.^13^, ^18^ Analysing and understanding these differences, especially in terms of age groups where hormonal transitions are occurring, might also provide some new insights into the pathomechanism of this complex disease.

To address these gaps, we conducted a large‐scale analysis using data from the Chronic Urticaria Registry (CURE)^19^ to investigate sex differences in disease activity, comorbidities, QoL, biomarkers, treatment responses, compliance to treatment and adverse events across different age groups of CSU patients.

## MATERIALS AND METHODS

### The CURE registry

CURE (https://www.urticaria‐registry.com/) is an ongoing, prospective, international, multicentre, observational, real‐world registry for CU and urticarial vasculitis patients. It was initially approved in 2014 by the local ethics committee of Charite—Universitätsmedizin Berlin (EA1/146/14). The design of data collection, development of CURE questionnaires and details for participating in the registry have been previously described.^19^, ^20^, ^21^

### Data collection and analysis

In CURE, data collection occurs at the patient's baseline visit and at least every 6 months (follow‐up). In this study, only baseline data were analysed (data cut: May 2023) and compared between female and male patients with CSU. These included patients’ demographics, age of onset, duration of urticaria, symptoms (wheals and/or angioedema), patient‐reported outcome measures (PROMs; Urticaria Activity Score over 7 days [UAS7], Urticaria Control Test [UCT], Chronic Urticaria Quality of Life Questionnaire [CU‐Q_2_oL] scores), family history of CSU, systemic symptoms (1. recurrent unexplained fever, 2. joint/bone/muscle pain, 3. malaise or 4. unknown), aggravating factors for urticaria (stress, food, infections and medications), comorbidities, smoking and alcohol consumption, laboratory parameters (leukocyte and C‐reactive protein [CRP] elevation), burden of disease (number of visits to hospital, number of hospital admissions, number of emergency visits, sleep disturbance [was your sleep disturbed by urticaria? Yes or no] and days missed from work or school),^3^ treatment distribution and response rates (poor disease control indicated by UCT < 12), treatment intolerance, low compliance and common side effects.^3^ Baseline age groups [<7, 7–12, 13–17, 18–30, 31–50, 51–65, 66–80, ≥81 years]) were recorded to identify the effects of hormonal changes, which roughly equate to the years of childhood, preadolescent, adolescent, young adult, child‐bearing age, menopause and elderly. However, when comparing the differences between females and males concerning age groups, five age groups were used: <13, 13–17, 18–30, 31–50, 51–65 and >65. UCT evaluated treatment responses.

### Definitions of sex

In this article, the patients' sex was determined by their physical and physiological features and recorded by the physicians rather than the patients' own definition of their gender. We preferred to use the terms ‘female’ and ‘male’ to describe patients' sex.

### Statistical analysis

Data were analysed using the Statistical Package for Social Sciences (SPSS) software, version 28.0. For descriptive analysis, frequency and percentage were used for categorical variables and mean and standard deviation were used for quantitative variables. The normality of continuous variables was confirmed using skewness, kurtosis and a visual inspection of the distribution histogram. For the bivariate analysis of continuous variables, the Student's *t*‐test was used to compare the means between the two groups after checking for homogeneity of variances using Levene's test; the Student corrected version was used in case of heterogeneous variances. A comparison of multiple subgroups was also conducted using ANOVA and adjusted for Bonferroni error. When comparisons were made between multiple subgroups, such as different age groups or treatment categories, each pairwise p‐value was multiplied by the total number of comparisons to determine statistical significance. The categorical variable association was assessed using the chi‐squared test. Afterwards, subgroup analysis according to age groups allowed for assessing the same associations in different categories. In all cases, a *p*‐value lower than 0.05 was considered significant.

For the multivariable analysis, logistic regressions were used to assess the correlates of poor disease control (UCT < 12); the model adequacy to the data was checked using the Hosmer–Lemeshow test. Independent variables introduced in the models were the ones considered of clinical importance, taking into account the allowed number of variables to be included given the sample size: sociodemographic and other independent variables were added as appropriate. Adjusted odds ratios and 95% confidence intervals (95% CI) were reported.

## RESULTS

### Female patients constitute Most of the CSU population

Of 4136 CSU patients from 58 participating sites from 29 countries (Table S1), females constituted 72.4% (*n* = 2994). The high rate of females compared to males visibly started at age 7 (Table 1); however, statistically significant female predominance (<0.001) started at age 31 (Table S2).

**TABLE 1:** Distribution of female and male CSU patients according to age groups.

Age group	Female (*n* = 2994), *n*, % (row)	Male (*n* = 1142), *n*, % (row)	Total (*n* = 4136), *n*, % (column)	*p* *
<7	5 (33.3)	10 (66.7)	15 (0.4)	<0.001
7–12	24 (52.2)	22 (47.8)	46 (1.1)	
13–17	50 (62.5)	30 (37.5)	80 (1.9)	
18–30	575 (73.2)	211 (26.8)	786 (19.0)	
31–50	1266 (73.0)	468 (27.0)	1734 (41.9)	
51–65	741 (72.1)	287 (27.9)	1028 (24.9)	
66–80	310 (74.2)	108 (25.8)	418 (10.1)	
≥81	23 (79.3)	6 (20.7)	29 (0.7)	
All ages	2994 (72.4)	1142 (27.6)	4136 (100)	

Abbreviations: CSU, chronic spontaneous urticaria; *n*, number of patients.

* *p*‐Value was derived from the chi‐squared test.

### Angioedema, a family history of CSU and systemic symptoms are more common in females than males

The frequency of angioedema (with or without wheals) was 65.0% in females versus 59.2% in males (*p* < 0.001) and 59.6% of the females vs 51.7% of the males presented with wheals and angioedema (*p* < 0.001). A family history of CSU (8.6% vs. 5.2%, *p* = 0.002) and systemic symptoms (fever, joint/bone/muscle pain and malaise) were more common among females than males (*p* < 0.001 for each; Table 2). Females more commonly implicated stress and food as aggravators of disease symptoms than males (23.6% vs. 18.3%, *p* < 0.001 and 14.1% vs. 11.6%, *p* = 0.04, respectively; Table S3).

**TABLE 2:** Comparison between female and male CSU patients concerning disease‐related features and burden of disease.

Features	Female (*n* = 2994)	Male (*n* = 1142)	Total (*n* = 4136)	p
Age in years (mean ± SD)	44.6 ± 16.1	43.3 ± 16.8	44.24 ± 16.3	0.018^a^
Age of onset (mean ± SD)	40.2 ± 16.7	39 ± 17.7	39.8 ± 17.0	NS^a^
Disease duration, years (mean ± SD)	4.44 ± 7.8	4.27 ± 8.5	4.39 ± 8.0	NS^a^
Angioedema, *n* (%)				
Only wheals	1026 (34.4)	457 (40.3)	1483 (36.0)	<0.001^b^
Wheals + angioedema	1777 (59.6)	586 (51.7)	2363 (57.4)	
Only angioedema	126 (4.2)	78 (6.9)	204 (5.0)	
Family history of CSU, *n* (%)	253 (8.6)	59 (5.2)	312 (7.6)	0.002^b^
Concomitant CIndU, *n* (%)	686 (23.2)	225 (19.7)	911 (22.4)	NS^b^
Systemic symptoms, *n* (%)				
Fever	123 (4.1)	38 (3.3)	161 (3.9)	<0.001^b^
Joint/bone/muscle pain	469 (15.7)	117 (10.2)	586 (14.2)	<0.001^b^
Malaise	443 (14.8)	135 (11.8)	578 (14.0)	<0.001^b^
Baseline CU‐Q_2_oL (mean ± SD)	32.1 ± 20.1	27.7 ± 19.1	30.9 ± 19.9	<0.001^a^
UCT < 12 baseline *n* (%)	1648 (72.8)	564 (68.3)	3090 (71.6)	0.010^b^
Sleep disturbance *n* (%)	1164 (38.9)	371 (32.5)	1535 (37.1)	<0.001^b^

Abbreviations: CIndU, chronic inducible urticaria; CSU, chronic spontaneous urticaria; CU‐Q_2_oL, chronic urticaria quality of life questionnaire; n, number of patients; NS, not significant; SD, standard deviation; UAS7, weekly urticaria activity score; UCT, urticaria control test; UCT < 12, poorly controlled urticaria.

^a^ The Student's test (or its corrected version when the variances were not homogeneous) was used.

^b^ The chi‐squared test was used.

### Most comorbidities are more frequent in females than males

Asthma, obesity, thyroid disease, autoimmune disease, gastrointestinal disease and depression were more frequent in females than in males (*p* < 0.05 for all). In contrast, males had a higher rate of diabetes (7.8% vs. 5.1%, *p* = 0.004), and smoking and alcohol consumption (12.2% vs. 7%, *p* < 0.001) compared to females (Table 3).

**TABLE 3:** Comorbidities and biomarkers in female and male patients with CSU.

Comorbidities	Female (*n* = 2994), *n* (%)	Male (*n* = 1142), *n* (%)	Total (*n* = 4136), *n* (%)	*p* *
Atopic dermatitis	144 (4.9)	53 (4.7)	197 (4.8)	NS
Allergic rhinitis	554 (18.8)	236 (20.9)	790 (19.4)	NS
Asthma	348 (11.8)	94 (8.3)	442 (10.9)	0.004
Food allergy	125 (4.5)	48 (4.5)	173 (4.5)	NS
Diabetes mellitus	150 (5.1)	88 (7.8)	238 (5.8)	0.004
Hypertension	554 (18.8)	221 (19.6)	775 (19.0)	NS
Hyperlipidaemia	308 (10.5)	144 (12.8)	452 (11.1)	NS
Obesity	416 (14.1)	127 (11.3)	543 (13.3)	0.048
Metabolic syndrome	74 (2.5)	19 (1.7)	93 (2.3)	NS
NSAID hypersensitivity	198 (6.7)	68 (6)	266 (6.5)	NS
Thyroid disease	583 (20.9)	72 (6.7)	655 (16.9)	<0.001
Autoimmune disease	360 (12.2)	53 (4.7)	413 (10.1)	<0.001
Celiac disease	8 (0.3)	2 (0.2)	10 (0.2)	NS
Myeloproliferative disease	6 (0.2)	3 (0.3)	9 (0.2)	NS
Gastrointestinal disease	609 (20.7)	172 (15.3)	781 (19.2)	<0.001
Depression	246 (8.4)	53 (4.7)	299 (7.3)	<0.001
Anxiety	339 (11.5)	105 (9.3)	444 (10.9)	NS
Toxic habit/drug abuse	205 (7.0)	138 (12.2)	343 (8.4)	<0.001
Elevated leukocytes	207 (9.7)	85 (10.2)	292 (9.8)	NS
Elevated CRP	498 (27.7)	180 (24.5)	678 (26.8)	NS

Abbreviations: CRP, C‐reactive protein; CSU, chronic spontaneous urticaria; *n*, number of patients; NS, not significant; SD, standard deviation; NSAID, non‐steroidal anti‐inflammatory drug.

* *p*‐Value was derived from the chi‐squared test.

### Quality of life impairment and sleep disturbance are higher and disease control is lower in females than in males

In females, the mean CU‐Q_2_oL score was higher (32.1 ± 20.1 vs. 27.7 ± 19.1, *p* < 0.001), more females rated their QoL as being ‘much’ affected by urticaria in the last 4 weeks (27.8% vs. 22.6%, *p* = 0.010) and sleep disturbance was higher compared to males (38.9% vs. 32.5%, *p* < 0.001, Table 2).

### Response to treatment is worse in females than in males

The rate of patients with poor disease control (UCT < 12) was higher in females than males globally (72.8% vs. 68.3%, *p* = 0.010; Table 2) and in each treatment category. Specifically, a higher number of females had UCT < 12 compared to males when treated with standard‐dose second‐generation anti‐histamines (sgAH; 70.5% vs. 66.9%, *p* = 0.02), high‐dose sgAH (76.5% vs. 73.9%, *p* = 0.02), omalizumab (53% vs. 44.5%, *p* = 0.03) and cyclosporine (81% vs. 71.4%, *p* = 0.02; Table 4). A multivariate analysis to determine risk factors for poorly controlled urticaria (UCT < 12) with variables sex, disease duration, concomitant CINDU, concomitant angioedema, NSAID hypersensitivity, thyroid disease, autoimmune disease, depression and body mass index revealed that being female increased the risk of poor urticaria control (UCT <12) in only patients aged 30–65. According to the analysis results, being female was associated with a 25% higher risk of poor control, and having concomitant CIndU increased the odds by 35%. Patients with depression had a marginally increased risk (0.1%; Table S4). A similar frequency of patients was treated with standard‐dose sgAH, high‐dose sgAH, omalizumab and cyclosporine. Intolerance to treatments, compliance with treatments and general side effects were similar; however, females reported more weight gain as a side effect with higher doses of sgAH (1.7% vs. 0.9%, *p* = 0.020; Table S5).

**TABLE 4:** Comparison of treatment response at baseline, compliance, intolerance and most common adverse events between females and males.

Treatment	Female (*n* = 2994) *n* (%)	Male (*n* = 1142) *n* (%)	Total (*n* = 4136) *n* (%)	*p* *
SgAH standard dose (*n* ^a^ = 1851)				
UCT < 12^b^	975 (70.5)	313 (66.9)	1288 (69.6)	0.02
Intolerance	151 (8.3)	48 (7.2)	199 (8.0)	NS
Low compliance	55 (3.0)	20 (3.0)	75 (3.0)	NS
Sedation	83 (4.6)	35 (5.2)	118 (4.7)	NS
Weight gain	16 (0.9)	2 (0.3)	18 (0.7)	NS
Headache	16 (0.9)	2 (0.3)	18 (0.7)	NS
SgAH high dose (*n* ^a^ = 1501)				
UCT < 12^b^	843 (76.5)	294 (73.9)	1137 (75.8)	0.02
Intolerance	122 (8.5)	45 (8.5)	167 (8.5)	NS
Low compliance	27 (1.9)	13 (2.5)	40 (2.0)	NS
Sedation	74 (5.1)	30 (5.7)	104 (5.3)	NS
Weight gain	24 (1.7)	5 (0.9)	29 (1.5)	0.02
Headache	15 (1.0)	4 (0.8)	19 (1.0)	NS
Omalizumab (*n* ^a^ = 474)				
UCT < 12^b^	188 (53.0)	52 (44.5)	240 (50.8)	0.03
Intolerance	50 (11.0)	18 (11.2)	68 (11.1)	NS
Low compliance	6 (1.3)	2 (2.1)	8 (1.3)	NS
Cyclosporine (*n* ^a^ = 47)				
UCT < 12^b^	17 (81.0)	7 (71.4)	22 (78.6)	0.02
Intolerance	2 (8.3)	0 (0.0)	2 (6.5)	NS

Abbreviations: *n*, number of patients; NS, not significant; sgAH, second‐generation antihistamine; UCT, urticaria control test; UCT < 12, poorly controlled urticaria.

^a^ Patients who received the mentioned treatment.

^b^ Only patients with UCT results. UCT<12 = poorly controlled urticaria.

* *p* Value was derived from the chi‐squared test.

### Females show higher disease burden than age‐matched males, especially between ages 51 and 65

**FIGURE 1:**
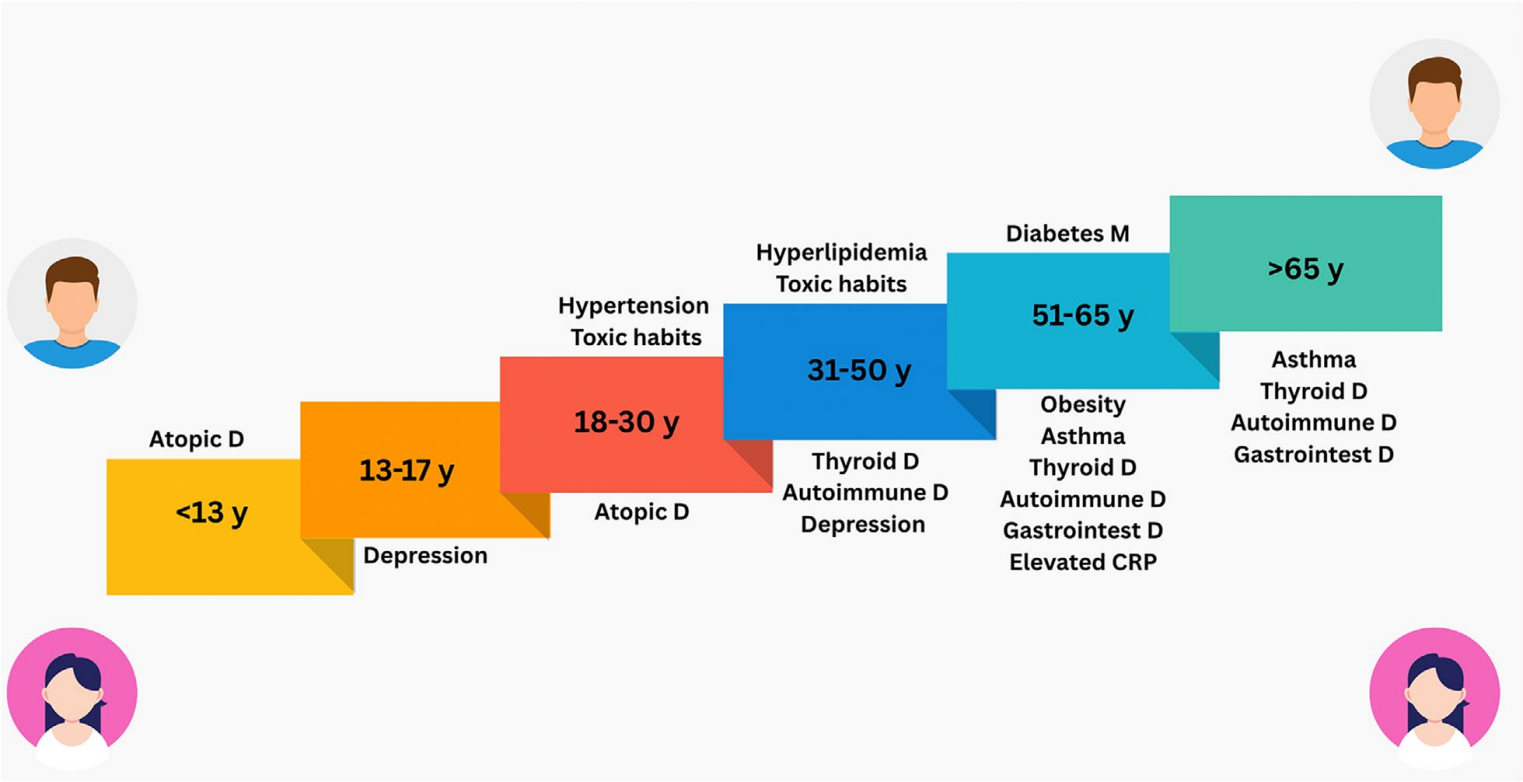
Comorbidities between female and male CSU patients according to age groups. CRP, C‐reactive protein; *n*, number of patients. Some parameters were significantly higher in females compared to males; the rates of comorbidities can be seen in Table S6.

**FIGURE 2:**
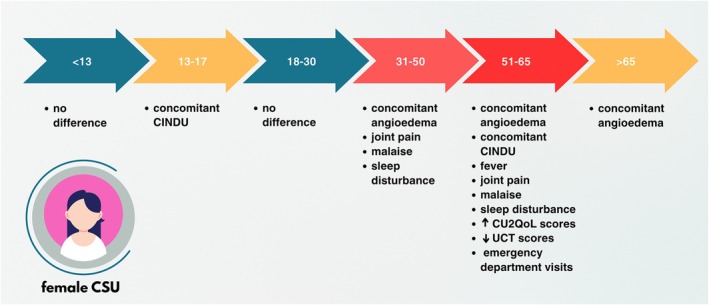
Burden of female CSU according to age groups compared to male CSU patients at the same age. AE, angioedema; CIndU, chronic inducible urticaria; *N*, number of patients; W, wheals. Some parameters were significantly higher in females compared to males; the rates of comorbidities can be seen in Table S6.

**FIGURE 3:**
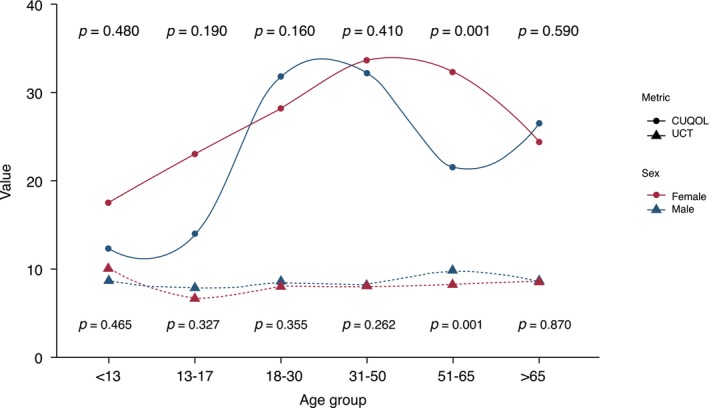
Baseline urticaria control test scores and CU‐_2_QoL scores for females and males across different age groups. CU‐2QoL scores and UCT scores were significantly higher and lower in female CSU compared to male CSU at ages 51‐65, respectively. The *x*‐axis shows the age group, and the *y*‐axis shows the value for the UCT or CU‐_2_QoL score. CU‐_2_QoL, Chronic Urticaria Quality of Life Questionnaire; *n*, number of patients; *p*, *p*‐value; UCT, urticaria control test. The *x*‐axis shows the age group, and the *y*‐axis shows the CU‐_2_QoL score.

The most prominent differences were observed at age 51–65 when female patients showed significantly higher frequencies of comorbid diseases and conditions (asthma, thyroid, autoimmune and gastrointestinal disease, obesity and elevated CRP; Table S6, Figure 1), angioedema, concomitant CIndU, systemic symptoms, sleep disturbance and emergency referrals (Figure 2), low baseline UCT (Figure 3), high CU‐_2_QoL scores (Figure 3; *p* < 0.05 for all comparisons; Table S7) than males.

Females aged 31–50, as compared to age‐matched males, showed significantly higher rates of angioedema, systemic symptoms and sleep disturbance, as well as more thyroid and autoimmune disease and depression. After age 65, only the rate of angioedema and comorbid diseases (asthma, thyroid, autoimmune and gastrointestinal disease) was higher in females compared to males (*p* < 0.05 for all comparisons; Tables S6 and S7, Figure 1). No differences in treatment responses were found across specific medication categories for different age groups.

## DISCUSSION

This CURE analysis showed a female predominance in CSU, which aligns with previous findings.^7^ Although a noticeable female predominance was observed from age 7 onwards, a statistically significant difference emerged only at age 31, with the female predominance continuing to increase beyond age 50. Even though this finding seems to contradict Fricke et al.^5^ who reported that females dominate CSU after age 15, Eun et al.^22^ found female predominance only for patients aged 20–64. This implies that, in addition to hormonal factors, age‐related changes in the immune system and increased inflammation may also contribute to the predominance of female patients in the CSU population.^23^ An intriguing, yet speculative, hypothesis is that fetal microchimerism—where cells from the fetus remain in the mother's body and may trigger a ‘graft‐versus‐host reaction’, potentially leading to autoimmune diseases—could contribute to the onset of CSU at reproductive age^23^; however, the effect of parity on CSU development is unknown and was not assessed in our study. Previous studies reported improvement in CSU during pregnancy^24^, ^25^ and worsening in the premenstrual period^26^ suggesting a relevant hormonal effect.

Female patients had a higher frequency of angioedema, more systemic symptoms, more sleep disturbance, higher CU‐Q2oL scores and a higher percentage of uncontrolled disease than males. Angioedema, systemic symptoms and sleep disturbance have been linked to more severe and uncontrolled disease as well as higher impairment in QoL, which all support that the impact of disease is higher in females compared to males.^20^, ^27^, ^28^, ^29^, ^30^ CSU's more burdensome and refractory presentation in females may be attributed to distinct pathomechanisms or the predominant endotype underlying the disease. We suggest that the greater burden of CSU in females may be due to a higher proportion of women exhibiting the Type IIb autoimmune endotype, which is characterized by a significant female predominance and typically presents with a more severe and refractory course compared to autoallergic CSU.^31^, ^32^, ^33^ However, we do not have markers, such as basophil tests, autologous serum skin tests (ASST) or IgG‐type autoantibodies, for autoimmune endotypes, for our patients.

We observed that CSU becomes even more burdensome for females in midlife (ages 51–65). Increases in disease severity and refractoriness in midlife may be attributed to the transition to menopause, due to changes in the immune system occurring at this time. Changes may include increased production and response to pro‐inflammatory cytokines and decreased secretion of anti‐inflammatory cytokines.^23^ These changes, coupled with altered endocrine function, may explain the increased burden of CSU in women with age. Additionally, the higher rates of thyroid and autoimmune diseases, obesity, CRP and systemic symptoms in females than in males in this age group support a multifactorial effect; hormonal, metabolic and immune changes lead to increased inflammatory cytokines and adipokines, thereby contributing to CSU by increasing inflammation and immune dysregulation.^23^

Of course, other potential contributors to the severity of disease in females could be responsible, including psychosocial stressors (e.g. caregiving roles, work stress and social determinants of health), differences in healthcare‐seeking behaviours and medication adherence. Examining these aspects individually and collecting patient data on each parameter would be interesting.

We found that concomitant conditions were more frequent in females than in males. These findings align with previous publications and underline the importance of a multisystemic approach to managing patients with CSU.^23^, ^34^, ^35^ We acknowledge that asthma (9.6% vs. 6.3%),^36^ thyroid disease,^37^ autoimmune disease,^38^ gastrointestinal disease^39^ and depression^40^ are often more common in females than in males in the general population. However, these comorbidities impose a disproportionately heavier burden on female CSU patients, amplifying their overall disease impact. Interestingly, although female patients develop a more refractory CSU phenotype after age 30 and become more burdened at ages 51–65 compared to males, after age 65, these differences mainly disappear. This, again, indicates the possible role of hormonal transitions in female disease processes. Prospective studies investigating hormonal and immunological profiles are needed to elucidate the underlying factors in disease manifestations across female age groups.

The overall response to urticaria treatments was poorer in females compared to males in every medication category, including sgAH, omalizumab and cyclosporine. After adjusting for various factors, being female was still a risk factor for poor urticaria control (UCT <12) in patients aged 30–65. Being female was associated with a 25% higher risk of poor control and having concomitant CIndU increased the odds by 35%, while depression added a marginally increased risk (0.1%). In contrast, a disease duration of less than 2 years was linked to a 23% reduction in the likelihood of poor control. These findings indicate that female sex is an independent risk factor for poor urticaria control in patients aged 30–65, even after adjusting for key clinical and comorbid variables, underscoring a potential biological or sex‐specific influence on disease severity and treatment response. The poorer response to urticaria treatment at this age in our study could be attributed to the higher occurrence of depression, autoimmunity, systemic inflammation (increased CRP levels), obesity and comorbid CIndU or the higher frequency of Type IIb autoimmune endotype in females.^27^, ^41^, ^42^, ^43^, ^44^, ^45^ However, the disparity in treatment response between sexes remains to be clarified.

Treatment compliance and side effects did not differ between sexes; however, weight gain was more commonly reported in females, especially with high‐dose sgAHs. This might be an important finding to consider while prescribing high‐dose sgAHs, especially in female patients who are anxious about gaining weight.

Differences in triggering factors between females and males for CSU have not been previously reported. The finding of stress and food as triggering factors could help female patients by informing them to try to manage or avoid stress if possible and make a food diary if they suspect any foods are exacerbating their symptoms.^46^

This study has several limitations. The small sample size in the younger patient group and the absence of UCT values for some patients limit the generalizability of the findings for these subgroups. Biomarker evaluation, such as basophil tests, ASST, autoantibodies, total IgE and anti‐thyroid peroxidase levels, would have been valuable for determining whether CSU in females adopts more Type IIb autoimmune patterns with age. This is particularly important as women over 50 tend to exhibit more refractory disease with increased systemic inflammation. The absence of hormonal profile data limits our ability to fully explore the role of hormonal transitions in the progression of CSU, especially the differences observed in male patients before puberty and female patients in menopause. Lastly, we did not assess other stressors, which could be contributing to the burden of female patients, such as caregiving roles and the impact of their work life.

Our findings show distinct disease patterns between female and male patients, which would encourage a patient‐tailored approach, that is, considering the higher burden of CSU in middle‐aged women (51–65 years) may help approach them more compassionately. The two most important findings of our study, female predominance in CSU starts after age 30 and CSU gets more burdensome in midlife, suggest there is a complex interplay between factors such as immunological differences, hormones and comorbidities in the severity and manifestation of the disease.

## CLINICAL RECOMMENDATIONS

Based on our findings, we recommend that female patients with CSU—particularly those aged 30–65—be routinely screened for comorbid conditions such as thyroid disorders, autoimmune diseases, depression, gastrointestinal conditions, asthma and obesity. Proactive identification and management of these comorbidities may help reduce the overall CSU disease burden. Additionally, it is important to recognize that female patients may respond less favourably to treatment compared to males. Therefore, timely escalation within the treatment algorithm should be considered to avoid unnecessary delays in achieving disease control.

In conclusion, female patients with CSU suffer far greater burdens from the disease than males, with more systemic symptoms, comorbidities, worse QoL, poorer disease control and responses to treatments. Our findings challenge the traditional notion that females overreport symptoms.^47^ Instead, we demonstrate a biological basis for these findings, supported by PROMs, laboratory findings and a higher prevalence of comorbid diseases. These findings highlight the need for tailored screening strategies, management and treatment approaches that address the unique challenges females with CSU face, ultimately improving their outcomes and QoL.

## AUTHOR CONTRIBUTIONS

All authors 1. Substantially contributed to the concept and/or design, or data acquisition, or data analysis and interpretation; 2. Drafted or critically revised the article for important intellectual content; and 3. Provided final approval of the version to be published.

## FUNDING INFORMATION

CURE funding sources are Novartis, Noucor, Sanofi and Moxie. However, this study has not received specific funding.

## CONFLICT OF INTEREST STATEMENT

**E Kocatürk** acted as a speaker for Novartis and Menarini. **P Salameh** has no conflict of interest to declare. **R Asero** is or recently was a speaker and/or advisor for Allergy Therapeutics, GlaxoSmithKline, HAL, Jasper, Malesci, Menarini, Novartis, Sanofi/Regeneron and Thermo‐Fisher. **M Bizjak** is or recently was a speaker and/or advisor for Novartis. **A Gimenez‐Arnau** is or recently was a speaker and/or advisor for and/or has received research funding from Almirall, Amgen, AstraZeneca, Avene, Blue‐Print, Celldex, Escient Pharmaceuticals, Genentech, GlaxoSmithKline, Harmonic Bio, Instituto Carlos III‐ FEDER, Jaspers, Leo Pharma, Menarini, Mitsubishi Tanabe Pharma, Novartis, Sanofi–Regeneron, Septerna, Servier, Thermo Fisher Scientific, Uriach Pharma and Noucor. **C Grattan** has no conflicts of interest. **D Pesqué** has received research grants from the Leo Foundation outside the submitted study. **L Shirin Herzog** has no conflicts of interest. **T Buttgereit** is or recently was a speaker and/or advisor for Almirall, Aquestive, BioCryst, CSL Behring, GlaxoSmithKline, Hexal, KalVista Pharmaceuticals, Medac, Novartis, Pharming, Roche, Sanofi‐Aventis, Swixx BioPharma and Takeda outside of the submitted work. **H Bonnekoh** was a speaker/consultant and/or advisor for and/or has received research funding from AbbVie, Celltrion, Novartis, Sanofi Aventis and ValenzaBio outside of submitted work. **D Fomina** has no conflicts of interest. **E Kovalkova** has no conflicts of interest. **M Lebedkina** has no conflicts of interest. **A Kasperska‐Zajac** has no conflicts of interest. **M Zając** has no conflicts of interest. **M Zamłyńsk** has no conflicts of interest. **K Kulthanan** has received educational speaker fees from Menarini, Novartis and Takeda. **P Tuchinda** has no conflicts of interest. **M Khouskhi** is a speaker and/or advisor for and/or has received research funding from Abidi Pharma, Alhavi Pharma, AstraZeneca, Ofogh Tolid Darou pars, GlaxoSmithKline and Danone, outside of submitted work. **Z Hassanpour** has no conflicts of interest. **J Peter** is or recently was a speaker and/or advisor and/or received educational grant support from Astria, AstraZeneca, CSL Behring, KalVista Pharmaceuticals, Novartis, Sanofi‐Regeneron, Glenmark Pharmaceuticals, Johnson and Johnson, Pharvaris and Takeda outside of the submitted work. **A Du‐Thanh** has no conflicts of interest. **R Meshkova** has no conflicts of interest. **M Abuzakouk** has no conflicts of interest. **M Makris** has no conflicts of interest. **L Bouillet** has consulted/served as a speaker for, engaged in research and educational projects with or accepted travel grants from the following companies: BioCryst, CSL Behring, Takeda, Novartis, GlaxoSmithKline, Blueprint, Intellia, Astra Zeneca, Pharvaris and Kalvista. **A Bocquet** has no conflicts of interest. **S Gregoriou** has no conflicts of interest. **S F Thomsen** is or recently was a speaker or advisor for AbbVie, Almirall, Boehringer, Eli Lilly, Galderma, Incyte, Janssen Pharmaceuticals, LEO Pharma, Novartis, Pfizer, Sanofi, UCB Pharma and Union Therapeutics, and received research support from AbbVie, Janssen Pharmaceuticals, LEO Pharma, Novartis, Sanofi and UCB Pharma outside the submitted work. **J Dissemond** has participated on advisory boards for Novartis. **P Staubach** has no conflicts of interest. **A Bauer** lectures and/or is an advisor for and/or has received project funding from Abbvie, Almirall, Amgen, AstraZeneca, Biofrontera, Celldex, Centogene, Escient, Galderma, Genentech, Incyte, Jasper, Leo, Lilly, Pharvaris, L'Oreal, Novartis, Regeneron, Sanofi, Shire and Takeda. **I Danilycheva** has no conflicts of interest. **M van Doorn** is or recently was a speaker and/or advisor for and/or has received research funding from Novartis, AbbVie, Almirall, Pfizer, LEO Pharma, Sanofi, Lilly, Janssen, UCB, BMS, GlaxoSmithKline, Maruho, Celgene, Third HarmonicBio and Escient. **C Parisi** has no conflicts of interest. **M Metz** is or recently was a speaker and/or advisor for AbbVie, Advanz, ALK‐Abello, Allegria, Almirall, Amgen, Argenx, AstraZeneca, Astria, Attovia, Berlin‐Chemie, Celldex, Celltrion, DeepApple, Escient, Ga2len, Galderma, GlaxoSmithKline, Incyte, Jasper, Lilly, Novartis, Pfizer, Pharvaris, Regeneron, Sanofi, Santa Ana Bio, Septerna, Teva, ThirdHarmonicBio and Vifor. **J Fluhr** has no conflicts of interest. **T Zuberbier. K Weller** is or recently was a speaker and/or advisor for and/or has received research funds from Moxie, Novartis, Pharvaris, Shire, Takeda and Uriach. **P Kolkhir** is or recently was a speaker and/or advisor for BioCryst, Novartis, Roche and ValenzaBio. Other authors have no conflict of interest to declare.

## ETHICAL APPROVAL

The Charité University Hospital of Berlin, Germany, Ethics Committee approved it (reference number EA1/146/14); all other participating centres obtained ethics committee approvals before joining the registry.

## ETHICS STATEMENT

This is not needed as this is a retrospective study. However, patients sign a consent form before entering their data into CURE.

## Supporting information


Data S1:

## Data Availability

The data supporting this study's findings are available from the corresponding author upon reasonable request.
