# Supplementary material for: Sex matters in CSU: Women face greater burden and poorer urticaria control, especially in midlife—CURE insights
Source: J Eur Acad Dermatol Venereol. 2025 Sep 18;40(1):67–78. doi: 10.1111/jdv.70027 (PMC12723574; doi:10.1111/jdv.70027)
Supplement: Supplementary file 1 — Data S1: [file JDV-40-67-s001.docx]

**Supplement**

**sTable 1: A List of Centers and Corresponding Entries/Patient Numbers used from Each Center for the Analysis**

| Center’s Name | Country | Number of Patients Included |
| --- | --- | --- |
| IAIS- Instituto de Alergia e Inmunología del Sur Bahía Blanca | Argentina | 6 |
| Hospital Italiano de Buenos Aires | Argentina | 72 |
| UCARE Center Allergy and Clinical Immunology of Federal University of Bahia - HUPES Federal University of Bahia Medical School Salvador | Brazil | 31 |
| Disciplina de Alergia Imunologia Clinica e Reumatologia of Departemento de Pediatria Universidade Federal de São Paulo | Brazil | 25 |
| Allergy out-patient clinic of Medical Centre "Doverie SBT" Sofia | Bulgaria | 3 |
| Department of Dermatology of Sun Yat-sen Memorial Hospital Sun Yat-sen University Guangzhou | China | 14 |
| Department of Dermatology of the Second Affiliated Hospital of Soochow University Suzhou | China | 48 |
| Group of Clinical and Experimental Allergy of IPS Universitaria Universidad de Antioquia Medellín | Colombia | 34 |
| Specijalna bolnica za plucne bolesti Zagreb | Croatia | 13 |
| Department of Dermato-Venereology and Wound Healing Centre of Bispebjerg Hospital Copenhagen | Denmark | 81 |
| RespiraLab Research Group Guayaquil | Ecuador | 3 |
| Internal Medicine Department National Reference Center for Angioedema (CREAK) of Grenoble Alpes University Hospital (CHUGA Grenoble) Grenoble | France | 88 |
| Unit of Allergy of Hospital Metz-Thionville Metz | France | 15 |
| Hospital CHU de Montpellier of University Hospital of Montpellier Saint-Eloi Montpellier | France | 101 |
| Assistance Publique-Hopitaux de Paris Service de dermatologie et allergologie of Hopital Tenon Paris | France | 24 |
| Institute of Allergology Berlin | Germany | 644 |
| Klinik für Dermatologie of Elbe Kliniken Buxtehude | Germany | 7 |
| Department of Dermatology and Allergology of Justus-Liebig-Universität Gießen | Germany | 6 |
| Department of Dermatology of University Hospital Essen | Germany | 71 |
| University Medical Center of Johannes Gutenberg-University Mainz | Germany | 74 |
| Department of Dermatology of Universitäts Allergie Centrum of Technische Universität Dresden Carl Gustav Carus Faculty of Medicine Dresden | Germany | 71 |
| Klinik für Hautkrankheiten of Universitätsklinikum Jena | Germany | 30 |
| Allergy Unit 'D. Kalogeromitros' 2nd Dpt. of Dermatology and Venereology Medical School National and Kapodistrian University of Athens University General Hospital 'ATTIKON' Athens | Greece | 88 |
| "Andreas Sygros" Hospital of National and Kapodistrian University of Athens | Greece | 93 |
| Allergy Dpt 2^nd^ Pediatric Clinic University of Athens | Greece | 13 |
| Allergy Clinic Preventive Medicine Unit of Kempegowda Institute of Medical Sciences (KIMS) and Research Center Bengaluru | India | 1 |
| Dr.D.Y.Patil University and School of Medicine Navi Mumbai | India | 17 |
| Allergy Research Center of Mashhad University of Medical Sciences Mashhad | Iran | 151 |
| Center for Research and Training in Skin Diseases and Leprosy of Teheran University of Medical Sciences Teheran | Iran | 22 |
| Central Pathology Laboratory of St. James's Hospital Dublin | Ireland | 7 |
| Ambulatorio di Allergologia of Clinica San Carlo Milan | Italy | 50 |
| Department of Dermatology of Hiroshima University Hospital Hiroshima | Japan | 26 |
| Division of Dermatology Department of Internal Related of Kobe University Graduate School of Medicine Kobe | Japan | 8 |
| Department of Dermatology of Yokohama City University Hospital Yokohama | Japan | 34 |
| Servicio de Alergia of Hospital Español de México Mexico City | Mexico | 3 |
| Erasmus University Medical Center Rotterdam | Netherlands | 63 |
| PHI University Clinic of Dermatology of University St. Cyril and Methodius Skopje | North Macedonia | 2 |
| Chair and Department of Dermatology Venereology and Pediatric Dermatology of Independent Public Clinical Hospital No. 1 Lublin | Poland | 30 |
| Allergology Unit of the Department of Dermatology Poznan of the University of Medical Sciences Poznan | Poland | 11 |
| Europ. Center for Diagnosis and Treatment of Urticaria Zabrze | Poland | 398 |
| Department of Dermatology of the University Hospital and Faculty of Medicine of the University of Coimbra | Portugal | 3 |
| Servico de Imunoloalergologia of Centro Hospitalar e Universitário de Coimbra | Portugal | 3 |
| Serviço de Imunoalergologia Centro Hospitalar De São João Porto | Portugal | 14 |
| Republican Clinical Hospital of Kazan State Medical University Kazan | Russia | 16 |
| NRC Institute of Immunology FMBA of Russia Republican Center of Clinical Immunology and Allergology Moscow | Russia | 69 |
| City Clinical Hospital № 52 of Moscow Ministry of Health Moscow | Russia | 634 |
| Department of Clinical Immunology and Allergy of Smolensk State Medical University Smolensk | Russia | 90 |
| King Khalid General Hospital Hafar Al Batin | Saudi Arabia | 2 |
| University Clinic of Respiratory and Allergic Diseases Golnik | Slovenia | 106 |
| Allergy & Immunology Unit (AIU) of University of Cape Town Lung Institute (Pty) Ltd Cape Town | South Africa | 128 |
| Hospital del Mar Research Institute Barcelona | Spain | 209 |
| Department of Dermatology Faculty of Medicine Siriraj hospital of Mahidol University Bangkok | Thailand | 169 |
| Department of Dermatology and Venereology Istanbul Faculty of Medicine of Istanbul University Istanbul | Turkey | 9 |
| Dermatology Clinic of Kayseri City Education and Research Hospital Kayseri | Turkey | 1 |
| Koc University School of Medicine Istanbul | Turkey | 46 |
| Department of Dermatology of Marmara University School of Medicine Istanbul | Turkey | 27 |
| Sakarya University Training and Research Hospital Sakarya | Turkey | 43 |
| Division Chair Allergy & Immunology of Cleveland Clinic Abu Dhabi | United Arab Emirates | 89 |

**sTable 2: Post Hoc Analysis for Female Percentage Among Age Groups**

| **Age groups** | | **Significance*** |
| --- | --- | --- |
|  |  |  |
| **<7 years** | 7–12 | NS |
|  | 13–17 | NS |
|  | 18–30 | NS |
|  | 31–50 | **0.038** |
|  | 51–65 | **0.037** |
|  | >65 | **0.015** |
| **7–12 Years** | <7 | NS |
|  | 13–17 | NS |
|  | 18–30 | NS |
|  | 31–50 | **0.039** |
|  | 51–65 | **0.039** |
|  | >65 | **0.009** |
| **13–17 years** | <7 | NS |
|  | 7–12 | NS |
|  | 18–30 | NS |
|  | 31–50 | NS |
|  | 51–65 | NS |
|  | >65 | **0.013** |
| **18–30 Years** | <7 | NS |
|  | 7–12 | NS |
|  | 13–17 | NS |
|  | 31–50 | NS |
|  | 51–65 | NS |
|  | >65 | NS |
| **31–50 years** | <7 | **0.038** |
|  | 7–12 | **0.039** |
|  | 13–17 | NS |
|  | 18–30 | NS |
|  | 51–65 | NS |
|  | >65 | NS |
| **51–65 years** | <7 | **0.037** |
|  | 7–12 | **0.039** |
|  | 13–17 | NS |
|  | 18–30 | NS |
|  | 31–50 | NS |
|  | >65 | NS |
| **>65 years** | <7 | **0.015** |
|  | 7–12 | **0.009** |
|  | 13–17 | **0.013** |
|  | 18–30 | NS |
|  | 31–50 | NS |
|  | 51–65 | NS |

sTable 1: Multivariate analysis for male percentage among age groups.

* P-value derived from post hoc testing with Bonferroni adjustment for multiple comparisons.

Confidence interval at the 0.05 level of the difference between percentages. NS, not significant.

**sTable 3: The Comparison Between Female and Male Patients Concerning Disease Aggravating Factors in CSU**

| **Factors** | **Female (n=2994)**  **n (%)** | **Male (n=1142)**  **n (%)** | **P*** |
| --- | --- | --- | --- |
| Infections | 157 (5.2) | 53 (4.6) | NS |
| Medication | 253 (8.5) | 89 (7.8) | NS |
| Major event | 15 (0.5) | 3 (0.3) | NS |
| Stress | 708 (23.6) | 209 (18.3) | **<0.001** |
| Food | 421 (14.1) | 133 (11.6) | **0.04** |
| Insect bite | 45 (1.5) | 10 (0.9) | NS |
| Systemic disease | 2 (0.1) | 0 (0) | NS |

*P-value was derived from the Chi-square test.

Abbreviations: n, number of patients; NS, not significant.

**sTable 4: Multivariate Analysis Results for Risk Factors for UCT<12 Between Ages 31–65**

| Variables | B | S.E. | Wald | df | Sig. | OR | 95% CI for EXP(B) | |
| --- | --- | --- | --- | --- | --- | --- | --- | --- |
|  |  |  |  |  |  |  | **Lower** | **Upper** |
| Gender male versus female | -0.291 | 0.115 | 6.419 | 1 | 0.011 | 0.747 | 0.597 | 0.936 |
| Disease duration >2 years | -0.263 | 0.104 | 6.407 | 1 | 0.011 | 0.768 | 0.627 | 0.942 |
| Additional CINDU | 0.298 | 0.123 | 5.855 | 1 | 0.016 | 1.347 | 1.058 | 1.714 |
| Wheals plus angioedema | 0.126 | 0.110 | 1.293 | 1 | 0.255 | 1.134 | 0.913 | 1.408 |
| NSAID hypersensitivity | 0.000 | 0.000 | 0.892 | 1 | 0.345 | 1.000 | 1.000 | 1.001 |
| Thyroid disease | 0.000 | 0.000 | 0.239 | 1 | NS | 1.000 | 0.999 | 1.001 |
| Autoimmune disease | 0.000 | 0.000 | 0.518 | 1 | 0.472 | 1.000 | 0.999 | 1.000 |
| Depression | 0.001 | 0.000 | 4.733 | 1 | 0.030 | 1.001 | 1.000 | 1.002 |
| BMI | 0.001 | 0.009 | 0.012 | 1 | NS | 1.001 | 0.983 | 1.019 |

NS, not significant.

**sTable 5: Comparison Concerning Comorbidities Among Age Groups Between Females and Males**

| Age | <13 y (n=61) | | | 13–17 y (n=80) | | | 18–30 y (n=786) | | | 31–50 y (n=1734) | | | 51–65 y (n=1028) | | | >65 y (n=447) | | |
| --- | --- | --- | --- | --- | --- | --- | --- | --- | --- | --- | --- | --- | --- | --- | --- | --- | --- | --- |
| Comorbidities | **Female**  **(n=29)** | **Male (n=32)** | **P*** | **Female (n=50)** | **Male (n=30)** | **P*** | **Female (n=575)** | **Male (n=211)** | **P*** | **Female (n=1266)** | **Male**  **(n=468)** | **P*** | **Female (n=741)** | **Male (n=287)** | **P*** | **Female (n=333)** | **Male (n=114)** | **P*** |
| Atopic dermatitis | 2 (6.9) | 10 (31.3) | **0.02** | 3 (6.4) | 3 (10.3) | NS | 58 (10.2) | 10 (4.9) | **0.02** | 49 (3.9) | 25 (5.2) | NS | 25 (3.4) | 3 (1.0) | NS | 7 (2.1) | 3 (2.7) | NS |
| Allergic rhinitis | 7 (24.1) | 10 (31.3) | NS | 8 (17) | 8 (27.6) | NS | 119 (21) | 47 (22.9) | NS | 257 (20.6) | 107 (23.2) | NS | 119 (16.3) | 54 (18.9) | NS | 44 (13.5) | 10 (8.8) | NS |
| Asthma | 3 (10.3) | 5 (15.6) | NS | 10 (21.3) | 4 (13.8) | NS | 60 (10.6) | 20 (9.8) | NS | 132 (10.6) | 37 (8) | NS | 98 (13.4) | 23 (8) | **0.02** | 45 (13.8) | 5 (4.4) | **0.02** |
| Food allergy | 0 (0) | 1 (3.2) | NS | 2 (4.7) | 1 (3.4) | NS | 32 (5.8) | 6 (3) | NS | 58 (4.9) | 30 (6.8) | NS | 24 (3.5) | 9 (3.4) | NS | 9 (3) | 1 (1) | NS |
| Diabetes mellitus | 0 (0) | 2 (6.3) | NS | 1 (2.1) | 0 | 0.05 | 10 (1.8) | 5 (2.4) | NS | 27 (2.2) | 14 (3) | NS | 60 (8.2) | 46 (16.1) | **0.01** | 52 (15.9) | 21 (18.6) | NS |
| Hypertension | 0 (0) | 0 (0) | - | 1 (2.1) | 2 (6.9) | NS | 2 (0.4) | 6 (2.9) | **0.01** | 101 (8.1) | 50 (10.8) | NS | 261 (35.7) | 100 (35) | NS | 190 (58) | 65 (57) | NS |
| Hyperlipidemia | 1 (3.4) | 0 (0) | NS | 1 (2.1) | 0 (0) | NS | 9 (1.6) | 5 (2.4) | NS | 78 (6.3) | 48 (10.4) | **0.008** | 135 (18.5) | 57 (19.9) | NS | 84 (25.7) | 34 (30.1) | NS |
| Obesity | 0 (0) | 0 (0) | - | 1 (2.1) | 0 (0) | NS | 27 (4.8) | 18 (8.8) | NS | 168 (13.5) | 56 (12.1) | NS | 154 (21.1) | 40 (14) | **0.02** | 66 (20.2) | 13 (11.5) | NS |
| Metabolic syndrome | 0 (0) | 0 (0) | - | 0 (0) | 0 (0) | - | 1 (0.2) | 0 (0) | NS | 12 (1) | 3 (0.6) | NS | 36 (4.9) | 10 (3.5) | NS | 25 (7.6) | 6 (5.3) | NS |
| NSAID hypersensitivity | 2 (6.9) | 1 (3.1) | NS | 4 (8.5) | 0 (0) | NS | 29 (5.1) | 7 (3.4) | NS | 87 (7) | 37 (8) | NS | 54 (7.4) | 17 (5.9) | NS | 22 (6.7) | 6 (5.3) | NS |
| Thyroid disease | 1 (3.4) | 2 (6.5) | NS | 2 (4.7) | 1 (3.4) | NS | 61 (11.1) | 12 (5.9) | NS | 234 (19.7) | 31 (7) | **<.001** | 187 (27.3) | 15 (5.6) | **<.001** | 98 (32.9) | 11 (10.5) | **<.001** |
| Autoimmune disease | 2 (6.9) | 3 (9.4) | NS | 1 (2.1) | 0 (0) | NS | 37 (6.5) | 6 (2.9) | NS | 147 (11.8) | 25 (5.4) | **<.001** | 117 (16) | 11 (3.8) | **<.001** | 56 (17.1) | 8 (7.1) | **0.03** |
| Celiac disease | 0 (0) | 1 (3.1) | NS | 1 (2.1) | 3 (10.3) | NS | 2 (0.4) | 0 (0) | NS | 5 (0.4) | 0 (0) | NS | 1 (0.1) | 2 (0.7) | NS | 0 (0) | 0 (0) | - |
| Myeloproliferative disease | 0 (0) | 0 (0) | - | 0 (0) | 0 (0) | - | 0 (0) | 0 (0) | - | 2 (0.2) | 1 (0.2) | NS | 2 (0.3) | 1 (0.3) | NS | 2 (0.6) | 1 (0.9) | NS |
| Gastrointestinal disease | 0 (0) | 3 (9.4) | NS | 2 (4.3) | 0 (0) | NS | 88 (15.5) | 30 (14.6) | NS | 251 (20.1) | 90 (19.5) | NS | 191 (26.1) | 36 (12.6) | **<.001** | 77 (23.5) | 13 (11.5) | **0.02** |
| Depression | 0 (0) | 1 (3.1) | NS | 5 (10.6) | 0 (0) | **0.04** | 35 (6.2) | 9 (4.4) | NS | 119 (9.6) | 26 (5.6) | **0.04** | 66 (9) | 14 (4.9) | NS | 21 (6.4) | 3 (2.7) | NS |
| Anxiety | 1  (3.4) | 2 (6.3) | NS | 6 (12.8) | 1 (3.4) | NS | 67 (11.8) | 24 (11.7) | NS | 159 (12.8) | 56 (12.1) | NS | 76 (10.4) | 18 (6.3) | NS | 30 (9.2) | 4 (3.5) | NS |
| Toxic habit/drug abuse | 0  (0) | 0 (0) | - | 0 (0) | 1 (3.4) | NS | 23  (4.1) | 25 (12.2) | **<.001** | 108  (8.7) | 75 (16.2) | **<.001** | 58  (7.9) | 28 (9.8) | NS | 16 (4.9) | 9 (8) | NS |
| Elevated leukocytes | 2 (10) | 0 (0) | NS | 0 (0) | 1 (4.5) | NS | 30 (7.8) | 15 (9.4) | NS | 96 (10.3) | 96 (10.5) | NS | 57 (10.5) | 22 (10.9) | NS | 22 (9.1) | 11 (13.4) | NS |
| Elevated CRP | 2 (13.3) | 4 (18.2) | NS | 3 (12) | 3 (17.6) | NS | 62 (19.4) | 25 (18.2) | NS | 209 (26.4) | 83 (26) | NS | 156 (34.1) | 45 (25.3) | **0.03** | 66 (34.7) | 20 (31.7) | NS |

*P-value was derived from the Chi-square test.

Abbreviations: CRP, C-reactive protein; n, number of patients; NS, not significant; NSAID, non-steroidal anti-inflammatory drug; y, years.

**sTable 6: Comparison Concerning CSU Features and Burden Among Age Groups Between Females and Males**

| Age | <13y (n=61) | | | 13–17y (n=80) | | | 18–30y (n=786) | | | 31–50y (n=1734) | | | 51–65y (n=1028) | | | >65y (n=447) | | |
| --- | --- | --- | --- | --- | --- | --- | --- | --- | --- | --- | --- | --- | --- | --- | --- | --- | --- | --- |
| Features | **Female (n=29)** | **Male (n=32)** | **P** | **Female (n=50)** | **Male (n=30)** | **p** | **Female (n=575)** | **Male (n=211)** | **p** | **Female (n=1266)** | **Male (n=468)** | **P** | **Female (n=741)** | **Male (n=287)** | **p** | **Female (n=333)** | **Male (n=114)** | **P** |
| Mean age (mean) | 8.4 | 8.3 | NS* | 15.5 | 15.2 | NS* | 25.2 | 24.9 | NS* | 40.4 | 39.8 | **0.03*** | 57.8 | 57.6 | NS* | 72.1 | 72.6 | NS* |
| Age of onset (mean) | 7.1 | 7.4 | NS* | 13.2 | 11.03 | NS* | 22.5 | 21.9 | NS* | 36.1 | 35.9 | NS* | 52.0 | 52.8 | NS* | 66.3 | 65.3 | NS* |
| Disease duration (mean) | 1.38 | 1.03 | NS* | 2.38 | 4.10 | NS* | 2.74 | 2.87 | NS* | 4.25 | 3.96 | NS* | 5.74 | 5.01 | NS* | 5.81 | 7.28 | NS* |
| Family history of CSU (n, %) | 3 (10.3) | 3 (9.4) | NS** | 11 (23.4) | 3 (10.3) | NS** | 59 (10.4) | 11 (5.4) | NS** | 103 (8.2) | 26 (5.6) | NS** | 57 (7.8) | 12 (4.2) | NS** | 20 (6.1) | 4 (3.5) | NS** |
| Angioedema (n, %)   - Only wheals - Wheals + angioedema - Only angioedema | 14 (48.3)  13 (44.8)  - | 14 (43.8)  18 (56.3)  - | NS** | 18 (37.5)  28 (58.3)  1 (2.1) | 14 (48.3)  15 (51.7)  0 (0) | NS** | 203 (35.6)  348 (60.9)  11 (1.9) | 84 (40.8)  112 (54.4)  8 (3.9) | NS** | 458 (36.3)  746 (59.1)  40 (3.2) | 197 (42.2)  254 (54.4)  10 (2.1) | **0.047**** | 212 (28.8)  472 (64)  41 (5.6) | 108 (37.6)  140 (48.8)  36 (12.5) | **<.001**** | 121 (36.3)  170 (51.1)  33 (9.9) | 40 (35.4)  47 (41.6)  24 (21.2) | **0.03**** |
| Concomitant CIndU (n, %) | 6 (20.7) | 8 (25) | NS** | 15(31.9) | 2 (6.9) | **0.02**** | 143(25.2) | 57 (27.3) | NS** | 322(25) | 105(22.4) | NS** | 158(21.6) | 42 (14.7) | **0.04**** | 42(12.8) | 12(10.7) | NS** |
| Systemic symptoms  (n, %)   - Fever - Joint/bone/muscle pain - Malaise | 0 (0)  0 (0)  1 (3.4) | 1 (3.1)  1 (3.1)  3 (9.4) | NS**  NS**  NS** | 4 (8)  11 (22)  9 (18) | 0 (0)  3 (10)  4 (13) | NS**  NS**  NS** | 23 (4)  85 (14.8)  95 (16.5) | 3 (1.4)  24 (11.4)  21 (10) | NS**  NS**  NS** | 56 (4.4)  208(16.4)  213(16.8) | 19 (4.1)  46 (9.8)  58 (12.4) | NS**  **<.001****  **0.02**** | 37 (5)  130(17.5)  96 (13) | 13 (4.5)  34 (11.8)  40 (13.9) | **0.04****  **0.01****  **0.03**** | 3 (0.9)  35 (10.5)  29 (8.7) | 2 (1.8)  9 (7.9)  9 (7.9) | NS**  NS**  NS** |
| Mean Baseline UAS7 | 8.77 | 18.13 | NS* | 24 | 13 | NS* | 17.02 | 17.27 | NS* | 18.6 | 17.6 | NS* | 15.89 | 15.05 | NS* | 16.45 | 14.98 | NS* |
| Mean CU-Q_2_oL score | 17.50 | 12.25 | NS* | 23 | 14 | NS* | 28.17 | 31.78 | NS* | 33.61 | 32.20 | NS* | 32.35 | 21.55 | **<0.001*** | 24.4 | 26.45 | NS* |
| Mean Baseline UCT | 10.11 | 8.67 | NS* | 6.71 | 7.76 | NS* | 7.99 | 8.36 | NS* | 7.97 | 8.29 | NS* | 8.24 | 9.69 | **<0.001*** | 8.61 | 8.51 | NS* |
| UCT <12 (n,%) | 5(55.6) | 12(66.7) | NS** | 33(86.8) | 16(76.2) | NS** | 335(75.1) | 125(74.9) | NS** | 740(73.8) | 247(69.6) | **0.04**** | 375(72.0) | 104(59.1) | **0.001**** | 171(65.3) | 61(67.8) | NS** |
| Sleep disturbance (n,%) | 8 (27.6) | 9 (28.1) | NS** | 19(43.2) | 9 (31) | NS** | 216 (39) | 69 (34.2) | NS** | 525(43.6) | 162(36.6) | **0.04**** | 293(42.2) | 92(34.8) | **0.010**** | 103(33.8) | 30(28.6) | NS** |
| Number of visits for CSU (mean) | N/A | N/A | - | N/A | N/A | - | N/A | N/A | - | 16.9 | 17.6 | NS* | 16.1 | 20.2 | NS* | N/A | N/A | - |
| Emergency dep visit (n,%) | 8 (27.6) | 7 (21.9) | NS** | 12 (25.5) | 3 (10.7) | NS** | 115 (20.6) | 40 (19.7) | NS** | 231 (18.8) | 70 (15.4) | NS** | 120 (16.6) | 44 (15.4) | **<.001**** | 54 (16.6) | 17 (15) | NS** |
| Missed any day at school/work yes (n,%) | 7 (24.1) | 7 (21.9) | NS** | 18 (38.3) | 9 (32.1) | NS** | 148 (26.5) | 60 (29.6) | NS** | 269 (21.9) | 96 (21.1) | NS** | 105 (14.5) | 40 (14) | NS** | 7 (2.2) | 3 (2.7) | NS** |
| Inpatient days (mean) | 5.25 | 8 | NS* | 12 | 5 | NS* | 6.34 | 5.90 | NS* | 6.4 | 6.2 | NS* | 6.61 | 5.18 | NS* | 7.36 | 4.17 | NS* |
| Systemic steroid use (n,%) | 2 (6.8) | 1 (3.1) | NS** | 1 (2.0) | 0 (0) | NS** | 29 (5.0) | 14 (6.6) | NS** | 100 (7.8) | 40 (8.5) | NS** | 56 (7.6) | 22 (7.6) | NS** | 24 (7.2) | 9 (7.9) | NS** |

*The Student test (or its corrected version when the variances were not homogeneous) was used.

**P-value was derived from the Chi-square test.

Abbreviations: CIndU, chronic inducible urticaria; CSU, chronic spontaneous urticaria; CU-Q_2_oL, chronic urticaria quality of life questionnaire; ICU, intensive care unit; n, number of patients; NS, not significant; SD, standard deviation; UAS7, weekly urticaria activity score; UCT, urticaria control test.
